# The Antiaging and Antioxidative Effects of a Combination of Resveratrol and High-Intensity Interval Training on the Frontal Lobe in Aged Rats: The Role of SIRTS 4, SIRTS 5, SOD1, and SOD2

**DOI:** 10.1155/omcl/8251896

**Published:** 2025-01-29

**Authors:** Amin Mehrabi, Reza Nuori, Abbasali Gaeini, Maryam Amirazodi, Mohammad Mehrtash, Mohsen Abedini Esfahlani, Mina Bahrami, Mohammad Abbas Bejeshk, Mohammad Amin Rajizadeh

**Affiliations:** ^1^Neuroscience Research Center, Institute of Neuropharmacology, Kerman University of Medical Sciences, Kerman, Iran; ^2^Department of Exercise Physiology, Kish International Campus, University of Tehran, Kish, Iran; ^3^Department of Exercise Physiology, University of Tehran, Tehran, Iran; ^4^Shiraz University International Division, Shiraz University, Shiraz, Iran; ^5^Faculty of Sport Science, Department of Exercise Physiology, Shahid Bahonar University, Kerman, Iran; ^6^Department of Tissue Engineering and Applied Cell Sciences, School of Advanced Technologies in Medicine, Tehran University of Medical Sciences, Tehran, Iran; ^7^Department of Exercise Physiology, Faculty of Physical Education and Exercise Sciences, Kerman Shahid Bahonar University, Kerman, Iran; ^8^Cardiovascular Research Center, Institute of Basic and Clinical Physiology Sciences, Kerman University of Medical Sciences, Kerman, Iran; ^9^Physiology Research Center, Institute of Neuropharmacology, Kerman University of Medical Sciences, Kerman, Iran

**Keywords:** aging, frontal lobe, HIIT, oxidative stress, resveratrol, sirtuins

## Abstract

**Introduction:** High-intensity interval training (HIIT) is a form of interval exercise that enhances capacity and benefits well-being. Resveratrol is a naturally occurring polyphenol prevalent in grapes and red wine, demonstrating significant health effects on the body. This study sought to evaluate the synergistic effects of swimming HIIT and resveratrol intake on the expression of SIRTs 4, SIRTs 5, and superoxide dismutases (SOD1 and SOD2) in the frontal lobe of elderly rats.

**Materials and Methods:** Forty-five male Wistar rats, aged 22 months, were categorized into five groups: the control group (CTL), the swimming HIIT group (Ex: Exercise), the swimming HIIT with resveratrol group (R + Ex), the resveratrol group (R), and the solvent control group (vehicle). The R + Ex group engaged in high-intensity interval swimming and ingested resveratrol (10 mg/kg/day via gavage) for 6 weeks. During the initial and final sessions of each week, blood samples from the rats in the Ex and R + Ex groups were collected for lactate analysis. The proteins SIRTs 4 and 5, as well as SODs 1 and 2, were quantified using the western blot approach.

**Results:** Integrating HIIT with resveratrol markedly enhanced the expression of SIRT4, SIRT5, and antioxidant enzymes in the frontal lobe of elderly rats.

**Conclusion:** Resveratrol and HIIT, particularly their synergistic effects, provide antioxidant and antiaging benefits on the frontal lobe of aged rats.

## 1. Introduction

Biologically, aging is caused by the cumulative effect of various cellular and molecular damages, resulting in a gentle reduction in physical and mental capacity, an elevated risk of malady, and, finally, death [1–3]. Aging increases the risk of diseases such as Alzheimer's disease, diabetes, cardiovascular diseases, stroke, and many others [4]. The global population of people over 65 years old is growing unprecedentedly, so it is expected that this population will reach 1.6 billion in the world by 2050 [5].

Oxidative stresses progressively accumulate over the lifespan, compromising mitochondrial function and causing damage to various bodily systems, especially the central nervous system [6]. The aging process and oxidative stress can impair the brain by adversely influencing neuroplasticity, brain homeostasis, and cognitive performance. In animal studies, glutathione shortage may hinder the aging brain's capacity to address oxidative stress, resulting in diminished physiological capabilities [7]. The aging process is characterized by diminished ATP generation, increased reactive oxygen species (ROS) production, and reduced antioxidant defenses within brain tissue. Increased amounts of ROS can induce oxidative stress and significant harm to cellular structures, including organelle membranes, DNA, lipids, and proteins within the brain [8, 9].

Exercise can help brain health through various molecular mechanisms, such as increasing the expression of BDNF, increasing the expression of proteins related to synaptic plasticity, and reducing oxidative stress, inflammation, and apoptosis [10]. The brain has high adaptability to physical activity, which causes morphological, metabolic, and functional changes following training [11–13]. Physical activity can affect the cognitive performance of the elderly and can also play an essential role in preventing neurodegenerative diseases [14, 15]. Meanwhile, there is restricted data about the exact molecular pathways that support the antioxidant impacts of training in the brain. However, it has been shown that long-term training reduces the amount of oxidants in the hippocampus of rats in the aging process, and physical activity increases the amount of GPX and SOD1 antioxidant enzymes [15–17].

Sirtuins (SIRT1-7) constitute a family of NAD+-dependent histone deacetylases. Recent studies have shown that sirtuins alter multiple physiological processes associated with redox and antioxidant signaling. SIRT5 safeguards the cell against oxidants. SIRT4 diminishes the production of ROS and possesses antioxidant functions [18]. Sirtuins have essential functions in the regulation of cerebral function throughout the aging process. Microglial activation, potentially due to diminished sirtuin activity, triggers sustained neuroinflammation, leading to synaptic impairment and neuronal demise [19]. Brain-specific superoxide dismutases (SODs) are known to play a crucial role in regulating redox equilibrium and safeguarding the brain from oxidative damage [20].

Researchers have indicated that certain regions of the brain are more impacted by aging than others. The frontal aging hypothesis posits that certain elderly individuals have disproportionate age-related alterations in frontal lobe brain structures and corresponding cognitive functions. This idea has received endorsement from multiple lines of evidence, encompassing neuropsychological, electrophysiological, neuroanatomical, and functional neuroimaging research [21–23].

The frontal lobe hypothesis is a significant concept in the cognitive neuroscience of aging, suggesting that cognitive impairments in older adults predominantly result from the physical and functional decline of the frontal lobes [24]. The aging of the prefrontal cortex leads to impairments in cognitive control, encompassing sustained attention, selective attention, inhibitory control, working memory, and multitasking capabilities [25].

On the other hand, it has been shown that the expression of sirtuins can be changed following aging in the frontal lobe [26, 27]. The frontal lobe of the brain is particularly susceptible to oxidative stress-induced damage, indicating a close relationship between the age-related loss in cognitive ability and heightened oxidative stress [28].

Furthermore, in our previous studies, we showed the protective impacts of swimming exercise and resveratrol on the hippocampus of aged rats [29, 30]. So, the evaluation of the frontal lobe is essential.

Herbal medicine can be a therapeutic strategy for some diseases, such as asthma [31, 32], diabetes [33], aging [29, 30, 34, 35], colitis [36], and hepatic encephalopathy [37].

Resveratrol is a member of the stilbenoid subgroup of polyphenols, characterized by two phenolic rings connected by an ethylene bridge. This natural polyphenol has been identified in over 70 plant species, particularly in the skins and seeds of grapes, and is present in limited quantities in red wines and diverse meals [38, 39]. Resveratrol exhibits anti-inflammatory, antioxidative, neuroprotective, and antiviral properties [40]. The neuroprotective impacts of resveratrol on the hippocampus and Alzheimer's disease in rats have been shown [41–43]. Our previous studies also revealed that resveratrol had neuroprotective impacts in the hippocampus of aged rats [29, 30, 34]. Resveratrol ameliorates well-being and contributes to longevity in many creatures [38]. Multiple targets and different mechanisms of action have been suggested to justify the beneficial impacts of resveratrol on health and longevity, including activating the sirtuins protein family [44]. Although some studies speculate that sirtuins are not related to longevity [45], many studies state that there is a strong relationship between sirtuins and longevity [46–50].

The positive impacts of a combination of other polyphenols, such as quercetin [51] and carvacrol [52] with exercise have been shown on neurodegenerative diseases. Besides, our previous research revealed the promising impacts of a combination of resveratrol and exercise on the hippocampus of aged rats [29, 30].

It has also been found that the SIRT5 gene and protein expression increase with resveratrol [53]. Resveratrol stimulates the enzyme activity and deacetylase of SIRT3 and SIRT4 to activate the downstream molecules [54].

Considering the challenge of population aging, the elderly's tendency towards swimming sports, the importance of sirtuins and SOD, the relationship of these proteins with aging, that brain cells are sensitive to mitochondrial dysfunction, and a relatively large number of reactive species are produced in their mitochondria. The current research is designed to evaluate the combined impact of very intense intermittent swimming training and resveratrol supplementation on sirtuins and mitochondrial SODs (SIRT4, SIRT5, SOD-1, and SOD-2) in the frontal brain lobe of old rats. Given that older people may not be able to exercise well due to physical conditions, it may be helpful to take a supplement along with exercise, significantly if it enhances the effects of exercise.

## 2. Materials and Methods

### 2.1. Animals

The Ethics Committee of the Kerman Neuroscience Research Center (Ethical code: KNRC/24-96/E) supervised all the laboratory interventions. Sixty (12-month-old) male Wistar rats (average: 325 g) were kept in a cage for 10 months and reached an average of 400 g. The animals were maintained under standard conditions. After the rats reached the appropriate age, 45 rats were selected for the main study. Sixty male rats, aged 10 months in the beginning, were bought and kept in the animal laboratory for 10 months. After 10 months and before starting the research, the 20-month-old rats underwent novel object recognition and open field tests, and finally, 45 rats with no movement disorders were included in the study. In our previous work, we demonstrated that aged rats show reduced recognition memory and elevated anxiety during novel object and open field tests, and this was an essential criterion for selecting the 45 animals evaluated in this study [34]. Many investigations have revealed that a 20-month-old rat is an aged rat [55, 56]; it has been shown that a 20-month-old rat is almost as old as a 60-year-old human [57]. The rats were allocated into five groups of nine each: (1) control group (CTL): rats with no intervention, (2) swimming high-intensity interval training (HIIT) (Ex: Exercise): rats that experienced exercise, (3) resveratrol administration (R): rats that received resveratrol orally by gavage (10 mg/kg) [29, 58], (4) swimming HIIT with resveratrol administration (R + Ex): rats that received resveratrol with exercise, and (5) solvent of resveratrol administration (rats that received methylcellulose). The concentration of resveratrol in the brain tissue will reach 43% of the oral administration dose after 2 h [59].

### 2.2. Exercise Protocol

The animals in the exercise group engaged in HIIT swimming, comprising 14 bouts of 20 s each, with a 10-s rest between every two sessions—the water temperature measured 26°C. The animals exercised thrice weekly (alternating days) for 6 weeks. In the initial week, the rats carried a weight corresponding to 9% of their body weight, with an additional 1% incrementally added each subsequent week; hence, by the sixth week, the rats bore a weight similar to 14%. The weight was affixed to the base of their tails. The R + Ex group was administered a resveratrol supplement (orally, 10 mg/kg) dissolved in 1% carboxymethyl cellulose with the exercise regimen. The R + Ex group engaged in HIIT swimming training alongside resveratrol solubilized in 1% carboxymethyl cellulose. Swimming occurred in the evening and under red illumination. The rats were administered resveratrol or carboxymethyl cellulose 2 h before the exercise. The R and R + EX groups were administered medications chronically before each exercise session, occurring three times weekly throughout 6 weeks [30]. 2 days following the final training session, the rats were subjected to mild anesthesia using a desiccator connected to a carbon dioxide capsule and were subsequently euthanized. Subsequently, the brains were dissected; the frontal lobe was excised from the entire brain, placed on ice, and preserved using liquid nitrogen.

### 2.3. Lactate Evaluation

In the first and third sessions of each week, the blood of the rats in the Ex and R + Ex groups was taken immediately from the tail vein for blood lactate measurement to evaluate the intensity of the exercise. Blood lactate was measured by a portable lactometer model EKF, Germany [30, 34].

### 2.4. Western Blotting

After the last session of exercise, the rats were killed with a lethal dose of ketamine (100 mg/kg) and xylazine (80 mg/kg) [60] and then the rats were decapitated, and the brain tissue was removed from their skulls. The Western blot approach assessed the expression levels of SIRT4, SIRT5, SOD1, and SOD2 proteins. The retrieved frontal lobe tissue weighed 40 μg and was normalized by weight across all animals. This method comprises three fundamental components. Initially, proteins are conveyed to the gel by electrophoresis. The proteins are transported to the polyvinylidene fluoride (PVDF) membrane in the subsequent stage. In the final phase, the proteins are recognized by a particular antibody. Subsequently, following the electrophoresis and protein separation from the gel, the proteins were transferred to the PVDF membrane. The subsequent stage involved blocking, followed by the documentation of the antibody–antigen combination with the enhanced chemiluminescence (ECL) technique and the gel dock apparatus. Following the digitization of the photos, band density was quantified with Image J software. The PVDF paper was immersed in the stripping solution at 55°C for 30 min. Following Tris-buffered saline with 0.1% Tween (TBST) washing, blocking was performed for beta actin [61].

### 2.5. Statistical Analysis

A one-way analysis of variance (ANOVA) was conducted in conjunction with Tukey's post hoc test. The significance level was less than 0.05. All data are shown as mean ± standard error of the mean (SEM). A two-way ANOVA with repeated measures assessed the blood lactate data.

## 3. Results

### 3.1. The Impact of HIIT and Resveratrol on Blood Lactate Levels

**Figure 1:**
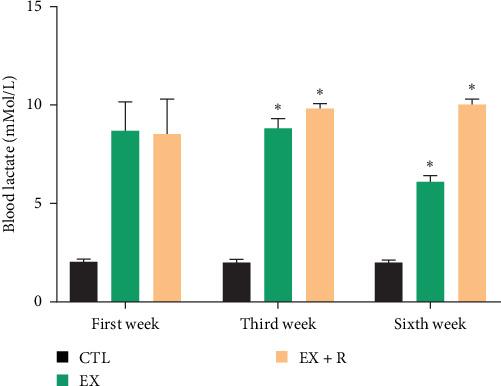
Mean alterations in blood lactate of aged rats in swimming HIIT in different weeks (mean ± SEM) (Ex, exercise; R, resveratrol). Two-way ANOVA with repeated measurement was used to analyze the data. *⁣*^*∗*^ Vs first week.

During the third week, blood lactate levels in R + Ex were markedly elevated compared to the first week. Furthermore, the blood lactate levels in the sixth week of the R + Ex group were considerably elevated compared to the first week. Our findings indicated that in the Ex group, blood lactate levels considerably diminished in the sixth week relative to the first week (Figure 1).

### 3.2. The Impacts of HIIT and Resveratrol on SIRT4 and SIRT5 Expression in the Frontal Lobe

**Figure 2:**
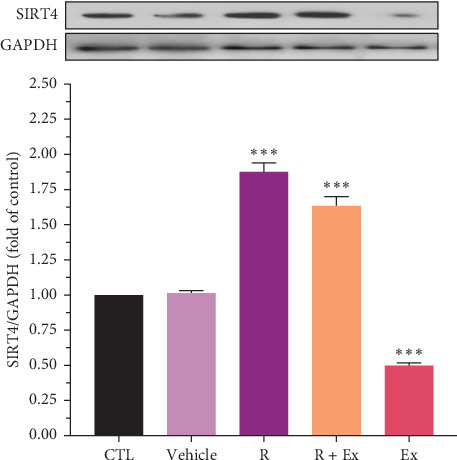
The impacts of resveratrol and HIIT and on SIRT4 expression in frontal lobe of aged rats. The data were presented as mean ± SEM, (*⁣*^*∗∗∗*^) *p*  < 0.001 compared to the CTL (old rats) group (*n* = 9 in each group). One-way ANOVA test was used for statistical analysis.

**Figure 3:**
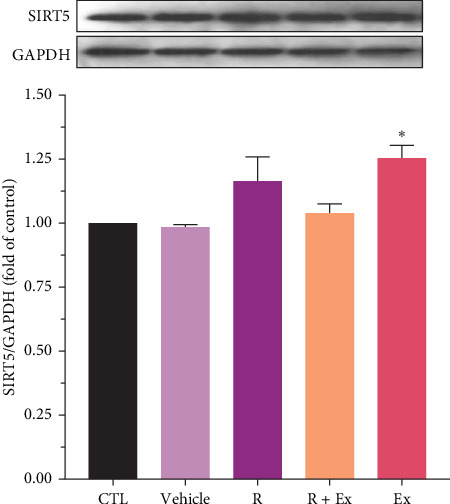
The impact of resveratrol and HIIT on SIRT5 expression in frontal lobe of aged rats. The data were presented as mean ± SEM, (*⁣*^*∗*^) *p*  < 0.05 compared to the CTL (old rats) group (*n* = 9 in each group). One-way ANOVA test was used for statistical analysis.

Resveratrol treatment significantly increased SIRT4 expression (1.87 ± 0.06, *p*  < 0.001) and had a nonsignificant effect on SIRT5 protein expression in the R group relative to the CTL group. Training significantly reduced SIRT4 expression (0.49 ± 0.01, *p*  < 0.001) and markedly raised SIRT5 protein expression (1.09 ± 0.009, *p*  < 0.05) in comparison to the CTL group. The combination of resveratrol and exercise significantly elevated SIRT4 expression (1.63 ± 0.06, *p*  < 0.001) compared to the control group. In the R + Ex group, the SIRT5 level was elevated compared to the control group although not considerably (Figures 2 and 3).

### 3.3. The Impact of HIIT and Resveratrol on the SOD1 and SOD 2 Expression in the Frontal Lobe

**Figure 4:**
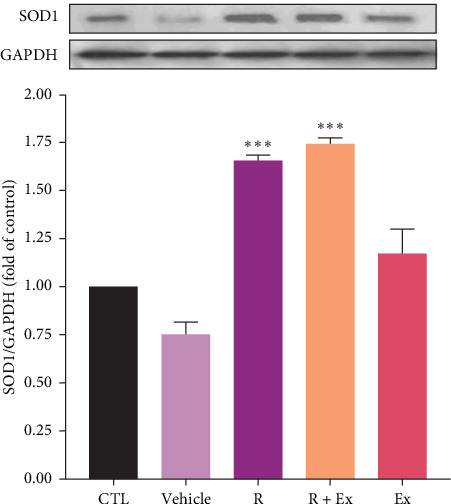
The impact of HIIT and resveratrol on SOD1 expression in frontal lobe of aged rats. The data were presented as mean ± SEM, (*⁣*^*∗∗∗*^) *p*  < 0.001 compared to the CTL (old rats) group (*n* = 9 in each group). One-way ANOVA test was used for statistical analysis.

**Figure 5:**
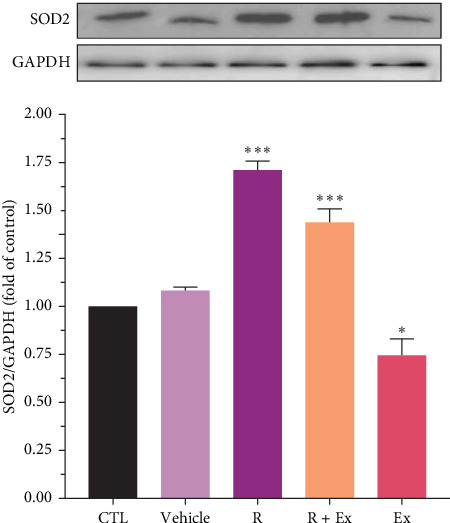
The impact of HIIT and resveratrol on SOD2 expression in the frontal lobe of aged rats. The data were presented as mean ± SEM, (*⁣*^*∗∗∗*^) *p*  < 0.001 and (*⁣*^*∗*^) *p*  < 0.05 compared to the CTL (old rats) group (*n* = 9 in each group). A one-way ANOVA test was used for statistical analysis.

Resveratrol significantly increased the expression of SOD 1 (1.65 ± 0.02, *p*  < 0.001) and SOD 2 (1.7 ± 0.04, *p*  < 0.001) proteins in the R group relative to the CTL group. The training increased SOD1 expression without a significant difference relative to the CTL group; however, SOD2 protein expression (0.74 ± 0.08, *p*  < 0.05) significantly decreased in the Ex group compared to the control group. The combination of resveratrol and HIIT significantly elevated the expressions of SOD 1 (1.74 ± 0.03, *p*  < 0.001) and SOD 2 (1.43 ± 0.07, *p*  < 0.001) in comparison to the CTL group (Figures 4 and 5).

## 4. Discussion

According to our observations, resveratrol increased the level of SIRT4 compared to the resveratrol-HIIT combination and HIIT swimming exercise alone. Moreover, since the HIIT swimming activity reduced the level of SIRT4, the interaction of resveratrol and HIIT swimming activity had a desirable effect on the level of SIRT4.

The blood lactate level decreased in the Ex group in the sixth week compared to the first week. The decrease in the average lactate level in the sixth week may be due to the adaptation created with intense training. The activity of lactate transporters (monocarboxylate transporters or MCT), which are responsible for transferring lactate from the blood into the cells, increases as a result of adaptation to exercise activity [62, 63].

Our findings also showed increased levels of lactate in the blood of animals in the Ex + R group. Since resveratrol is known to have exercise-mimetic effects, it seems to increase the intensity of exercise [64, 65]. Since resveratrol probably worked in parallel with intense exercise, a large amount of lactate was produced during training, and the disproportion between the performance of MCTs and the amount of lactate produced led to the delayed transfer of lactate from the blood into the cells. It seems that more time is needed to adapt to exercise and decrease the lactate trend, and if this study were to continue for 8 weeks, the decreasing trend of lactate would have probably been observed in this group.

The reduced level of SIRT4 caused by HIIT swimming exercise is consistent with the observations of Yaqub Nezhad et al. [66] They found that SIRT4 gene expression decreased after endurance training. Braidy et al. [26] verified that SIRT4 expression decreased in the frontal lobe tissue more than the other brain sections, such as the hippocampus and temporal lobe, following aging. Thus, it is possible that HIIT could not increase SIRT4 toward the normal range due to its intense reduction following aging. Furthermore, Hart et al. [67] revealed that 12 weeks of exercise training on a treadmill and treatment with resveratrol in 13-month-old male rats, which were low-capacity runners (LCR), remarkably reduced SIRT4 levels. These findings are in line with the data of the current research. The period of long-term exercise probably decreased SIRT4 levels. Moreover, the intensity of the exercises in the training program of the present study (14 times, each lasting 20 s in each session) was relatively high, and energy production methods that produce energy without the presence of oxygen were probably used to continue energy production (expect opening the anabolic window). Since SIRT4 plays an essential role in regulating the use of fatty acids, and the training program of this study was of the HIIT type, the rest intervals between training sets probably did not allow the initiation of the signaling pathways that activated SIRT4 [68]. Moreover, the type of rest between training sessions also leads to different results. Whether active rest or passive rest is used in the rest intervals between training sessions may affect the amount of SIRT4 protein in the frontal lobe. In addition, Karvinen et al. [69] demonstrated that the amount of SIRT4 protein in the skeletal muscle of rats did not change after voluntary running on a rotating wheel for 1 year. However, Radak et al. [70] found that SIRT4 expression in the skeletal muscle of mountaineers elevated after 5 weeks of being at altitude and climbing a height above 8000 m. These findings, which are not consistent with the outcomes of the present research, indicate that various interventions can affect the amount of SIRT4 in different tissues, leading to different outcomes and responses in the amount of SIRT4 protein. Increasing SIRT4 levels at high altitudes was likely caused by increased physical activity and fat metabolism. In this research, the amount of this protein decreased due to the intense nature of HIIT exercises. This is induced by the presence of rest intervals and metabolic turbulence caused by intense training sets of SIRT4 negative regulation. In the current research, the HIIT swimming exercise decreased the SIRT4 level; therefore, the HIIT swimming exercise is probably a negative regulator of SIRT4. Unfortunately, most of the studies in the literature have scrutinized SIRT4 in muscle tissues, and more studies should be conducted to investigate SIRT4 in nerve tissues.

Yokokawa et al. [71] reported that long-term running increased SIRT5 gene expression. Moreover, Karvinen et al. [69] declared that voluntary running on a rotating wheel does not change the amount of SIRT5. The findings of the current research are not in line with the findings of Karvinen et al. [69]. In this study, HIIT swimming exercise could not create the necessary arousal and stimulation to increase the amount of SIRT5; this outcome was probably due to the difference in the type of subjects since the subjects of the present study were old rats (24 months old), while Karvinen et al. [69] used younger rats (21 months old). However, given that running on a rotating wheel did not increase the level of SIRT5, age is probably an effective factor in the variations in the level of this protein; despite the high intensity of the exercises in this study, we still did not observe any increase in the amount of this protein. Another point that should be considered when scrutinizing the similarity of the results of the two studies is the amount of this protein in the skeletal muscle tissue [69] and the brain frontal lobe tissue (the present study). As mentioned previously, SIRT5 is a protein that plays an essential role in many processes, chemical reactions, and energy production cycles [72]. Based on the role of SIRT5, its downstream targets in the brain, and the significant increase in the amount of SIRT5 in the frontal lobe tissue of old rats after 6 weeks of HIIT swimming can be declared that the upstream regulation of SIRT5 better excretes ammonia through the glutamate–glutamine cycle and improves age-related decreased detoxification [73].

Our results demonstrated a reduction in SOD1 expression in the prefrontal cortex of aged rats. The administration of resveratrol, both independently and in conjunction with exercise, enhanced its expression, whereas exercise alone did not significantly affect the expression of SOD1. Moreover, our data revealed that resveratrol treatment, both independently and in conjunction with exercise, elevated SOD2 expression, but exercise alone reduced SOD 2 expression.

Investigations have revealed the antioxidant impacts of resveratrol through increasing the expression of SOD1 and SOD2 [74, 75]. According to the oxidative stress theory of aging, investigations revealed the reduced expression of antioxidant agents, such as SOD, in old animals [30, 76]. Our findings also disclosed that the expression of SODs diminished in old rats, and resveratrol alone increased their expression. In other words, resveratrol acted to reduce oxidative stress by increasing the expression of SODs.

Our results showed that exercise alone decreased SOD2 expression and had no significant effect on SOD1 expression. This part of our findings is somewhat controversial. Some investigations have revealed that exercise elevates the expression of SOD, which is contrary to our findings [16, 77]. Furthermore, our previous study demonstrated similar findings in the hippocampus of old rats [30]. Our findings indicate that acute episodes of prolonged, high-intensity endurance exercise in untrained humans and animals elevate biomarkers of oxidative stress, including a drop in enzymatic antioxidants [78, 79].The differences in the data can be owing to the type of training, the type of rodent and its species, the age and sex of the animal model, and the area of the brain studied.

The primary objective of this study was to examine the synergistic effect of exercise and resveratrol. Our results indicated that this combination enhanced the expression of SOD1 and SOD2. Resveratrol, as an exercise mimetic agent, augmented the antioxidant effects of physical activity.

Resveratrol increased the storage of ROS in cells and caused the transcription of diverse antioxidant genes. Hence, even oxidative stress due to small amounts of resveratrol can present a response such that the cells can adapt properly and avoid the damage resulting from oxidative stress. The activation of mitochondrial sirtuin modulators through regulating the ratio of NAD^+^/NADH or the activation of AMPK-dependent pathways, involved in the main mechanism of resveratrol effects in cells, is likely to create antioxidant balance in cells.

The combination of other polyphenols, such as blueberry [80], grape seeds [81], and green tea [82], with exercise has neuroprotective effects through reducing inflammation, oxidative stress, and apoptosis and modulating neurogenesis.

This research showed for the first time the interaction between SIRTT4 and SIRT5 and antioxidant enzymes (SOD1 and SOD2) in the frontal lobe under the influence of resveratrol and HIIT following aging. It seems that resveratrol and its combination with HIIT improve the condition of the frontal lobe following aging by increasing the expression of SIRT 4 and 5 and antioxidants enzymes (SOD 1 and SOD 2) and modulating their interaction.

Based on our results, HIIT and resveratrol enhanced each other's effects on some factors. It seems that one mechanism of this synergistic effect is the combined effects of HIIT and resveratrol on mitochondrial proteins such as pyruvate dehydrogenase E1 subunit alpha 1 and NAD+, which we mentioned in our previous study [29]. Rashet, Abdi, and Barari [83] elucidated the synergistic effect of aerobic training and resveratrol on the AMPK/PGC1-*α*/SIRT1 pathway in the hippocampus of rats afflicted with Alzheimer's Disease.

## 5. Conclusion

Resveratrol increased the expression of SOD1, SOD2, SIRT4, and SIRT5 in the frontal lobe of aged rats. Resveratrol alone seemed to exert protective effects. Regarding HIIT, our observations showed that HIIT increased the expression of SOD1 and SIRT5 but decreased the expression of SOD2 and SIRT4 in the frontal lobe of aged rats. Based on our results, the effects of HIIT alone are controversial. We suggest that it is better to evaluate other SIRTs (such as SIRT1) or other oxidative stress parameters (such as MDA) to elucidate this matter in future studies. However, the main goal of this study was to assess the combination of resveratrol and HIIT. It seems that the combination of resveratrol and HIIT was more effective in modulating SIRTs and SODs in the frontal lobe of aged rats.

## 6. Limitations

Due to financial limitations, we could not measure more SIRTs or oxidative stress-related parameters and pathways. We recommend that future studies assess some behavioral functions.

## Data Availability

The data will be available at a reasonable request.
